# Effects of environmental modification on the diversity and positivity of anopheline mosquito aquatic habitats at Arjo-Dedessa irrigation development site, Southwest Ethiopia

**DOI:** 10.1186/s40249-019-0620-y

**Published:** 2020-01-27

**Authors:** Dawit Hawaria, Assalif Demissew, Solomon Kibret, Ming-Chieh Lee, Delenasaw Yewhalaw, Guiyun Yan

**Affiliations:** 1 Yirgalem Hospital Medical College, Yirgalem, Ethiopia; 2 Department of Medical Laboratory Sciences and Pathology, Institute of Health, Jimma University, Jimma, Ethiopia; 3 Department of Medical Laboratory Sciences, College of Medicine and Health Sciences, Ambo University, Ambo, Ethiopia; 4 Aklilu Lemma Institute of Pathobiology, Addis Ababa University, Addis Ababa, Ethiopia; 5 Program in Public Health, University of California at Irvine, Irvine, CA 92697 USA; 6 Tropical and Infectious Diseases Research Center (TIDRC), Jimma University, Jimma, Ethiopia

**Keywords:** Anopheline mosquito breeding, Mosquito habitat, Malaria, Irrigation, Ethiopia

## Abstract

**Background:**

Irrigated agriculture is key to increase agricultural productivity and ensure food security in Africa. However, unintended negative public health impacts (e.g. malaria) of such environmental modification have been a challenge. This study assessed the diversity and distribution of breeding habitats of malaria vector mosquitoes around Arjo-Dedessa irrigation development site in Southwest Ethiopia.

**Methods:**

Anopheline mosquito larvae were surveyed from two agroecosystems, ‘irrigated’ and ‘non-irrigated’ areas during the dry (December 2017–February 2018) and wet (June 2018–August 2018) seasons. Mosquito habitat diversity and larval abundance were compared between the irrigated and non-irrigated areas. The association between anopheline mosquito larvae occurrence and environmental parameters was analysed using Pearson chi-square. Multiple logistic regression analysis was used to determine primary parameters that influence the occurrence of anopheline larvae.

**Results:**

Overall, 319 aquatic habitats were surveyed during the study period. Around 60% (*n* = 152) of the habitats were positive for anopheline mosquito larvae, of which 63.8% (*n* = 97) and 36.2% (*n* = 55) were from irrigated and non-irrigated areas, respectively. The number of anopheline positive habitats was two-fold higher in irrigated than non-irrigated areas. Anopheline larval abundance in the irrigated area was 16.6% higher than the non-irrigated area. Pearson’s chi-square analysis showed that season (*χ*^2^ = 63.122, df = 1, *P* < 0.001), agroecosystem (being irrigated or non-irrigated) (*χ*^2^ = 6.448, df = 1, *P* = 0.011), and turbidity (*χ*^2^ = 7.296, df = 2, *P* = 0.025) had a significant association with larval anopheline occurrence.

**Conclusions:**

The study showed a higher anopheline mosquito breeding habitat diversity, larval occurrence and abundance in the irrigated than non-irrigated areas in both dry and wet seasons. This indicates that irrigation development activities contribute to proliferation of suitable mosquito breeding habitats that could increase the risk of malaria transmission. Incorporating larval source management into routine malaria vector control strategies could help reduce mosquito population density and malaria transmission around irrigation schemes.

## Background

Irrigation schemes are key to increase agricultural productivity, ensure food security, promote economic growth and alleviating poverty in the developing world [1]. However, past experience shows that inadequate consideration of the impact of environmental modification on the distribution of vector-borne diseases could lead to public health challenges [2]. Malaria is one of the major public health challenges that occurs around irrigation schemes in Africa [3, 4].

The distribution of malaria is mainly governed by the spatial and temporal distribution of malaria vectors in different ecological settings. Environmental modifications such as construction of irrigation schemes could alter the existing ecological setting and favor breeding of mosquitoes by providing additional aquatic habitats [5]. Such environmental changes may also lead to the change in mosquito vector diversity, distribution, abundance and proliferation. Studies are thus required to understand the dynamics of mosquito breeding habitats that can be created due to environmental modifications. Identifying the source of mosquitoes helps decision makers to implement tailor-made mosquito vector interventions.

In Ethiopia, malaria is the leading public health problem and 68% of the population lives in malarious areas [6]. Although more than 42 species of *Anopheles* mosquitoes have been documented, *An. arabiensis* is the most widely distributed primary vector of malaria in the country [7]. The major malaria vector control strategies encompasses use of long-lasting insecticidal nets (LLIN), indoor residual spraying (IRS); and artemisinin-based combination therapy treatment [8].

In recent years, Ethiopia has seen an extensive irrigation development aimed to improve its crop production and promote economic growth [9]. The impact of such large scale water resources development schemes on malaria risk, however, has been poorly studied. As the country is striving to eliminate malaria from endemic areas by 2030 [10], it is important to identify risk factors associated with malaria in different settings. Understanding malaria vector mosquitoes larval ecology, diversity and distribution is therefore crucial in order to devise intervention measures [11–13].

This study aims to assess the impact of large scale irrigation on the malaria vector mosquitoes larval breeding and abundance. It evaluates how irrigated areas affect availability of positive larval habitats as compared to non-irrigated areas. Furthermore, the study describes the major breeding habitats of anopheline mosquitoes in the area.

## Methods

### Study setting

**Fig. 1:**
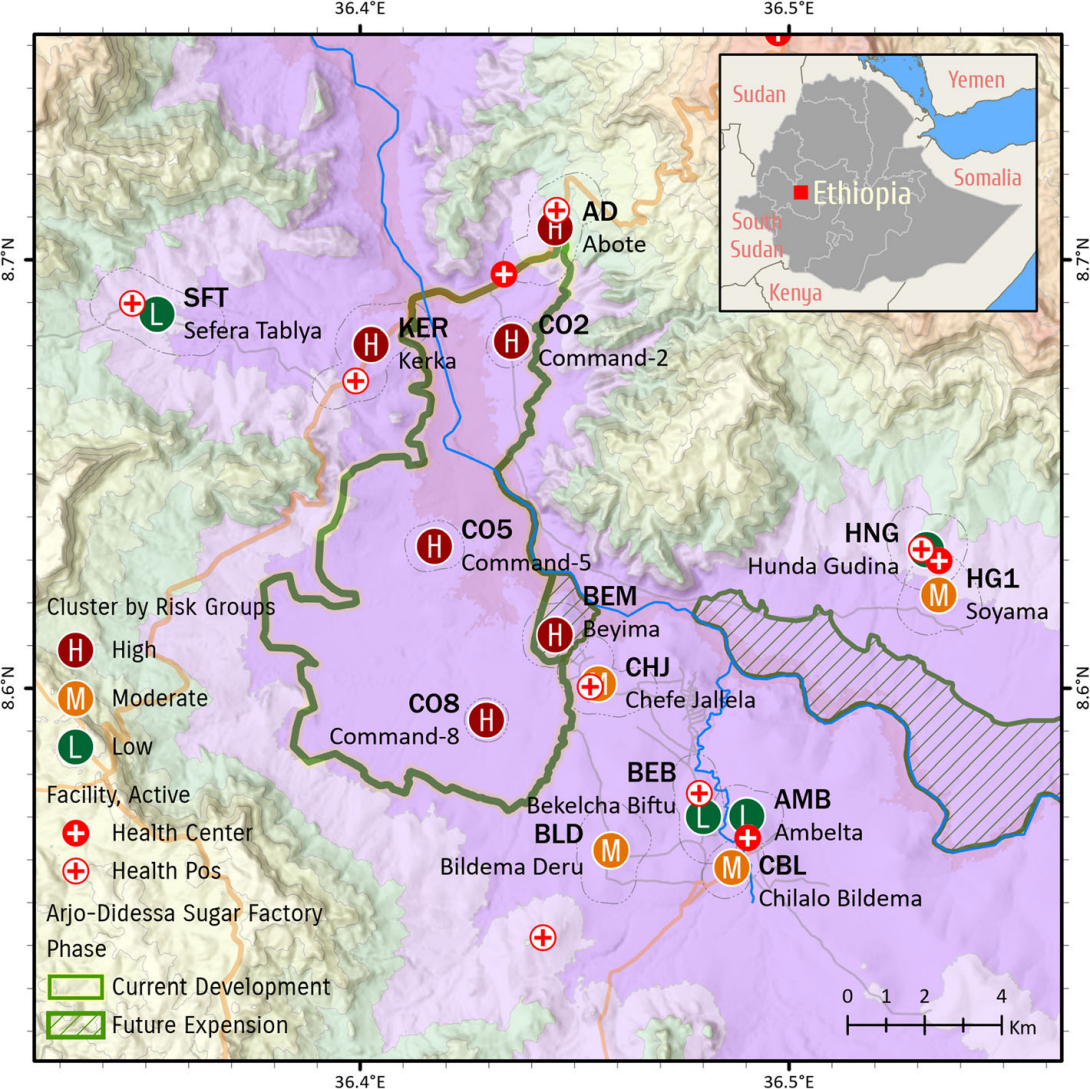
Site map of the study area

The study was conducted at Arjo-Dedessa irrigation development site and its vicinity located in Southwest Ethiopia (Fig. 1). Arjo-Dedessa irrigation development site is one of the largest projects in the country. Historically, the area was a wildlife sanctuary called ‘Dedessa wildlife sanctuary’, known by its thick forest. The large scale sugarcane plantation farm was established in 2006. Currently, the irrigation farm covers about 4000 ha of land, with an expansion plan to reach 80 000 ha in the next ten years. The irrigation scheme pumps water from Dedesa River, one of the major tributaries of the Blue Nile River basin. The total population in the study site was estimated to be 50 000. The altitude of the area ranges from 1300 to 2280 m above sea level with mean annual rainfall of 1477 mm.

The area is endemic to malaria [14] and *Plasmodium falciparum* is the dominant malaria parasite. While LLIN and IRS were routine practiced, larval habitat management through community involvement was rarely applied except during epidemic years.

Local communities in the area are depend on subsistence farming. They practice smallholder non-irrigated cultivation of mixed crops and cereals. The common crops cultivated in the area include, corn, maize, peanut, sorghum, rice, wheat and coffee. In addition to the Dedesa River, seasonal streams and springs are abundant in the area.

### Study period and design

A cross-sectional study was applied to assess the effect of irrigation activities on anopheline mosquitos’ larval habitat diversity and distribution. Larval abundance in two different agroecosystems, irrigated and non-irrigated areas, were compared. The study was conducted during the dry (December 2017–February 2018) and wet (June 2018–August 2018) seasons.

### Sampling site selection

The study site was first classified into ‘irrigated’ and ‘non-irrigated’ areas. Irrigated areas were considered as ‘risk’ areas for malaria and constituted irrigated farms and their surroundings within 1 km radius. Non-irrigated areas were those with low risk of malaria located outside the irrigation farms between 2 and 5 km from the irrigation schemes. These areas were further classified into clusters (a village with 100–150 households) and twelve clusters (six from irrigated and six from non-irrigated area) were selected and surveyed for aquatic larval breeding habitats.

### Larval survey

All accessible potential mosquito breeding habitats (i.e. any water containing structure) were surveyed for mosquito larvae. The larval surveys were conducted thoroughly within the estimated 1 km radius distance from the center of each cluster. Mosquito larvae were sampled following the WHO standard larval survey procedure using a standard dipper (350 ml, Bio Quip Products, Inc. California, USA) [15]. For larger breeding habitats, presence and absence of the larvae were determined after 20 dips. For habitats which were too small, dipping was done using pipettes. Water sampled by dipper was poured into a white sorting tray and checked for mosquito larvae. Larvae were identified morphologically and sorted by genus as *Anopheles* and *Culex*. Anopheline larvae were further sorted into species, and the corresponding counts were recorded. All anopheline larvae samples were poured into a plastic container and transported to the field insectary to rear them to adult stage for morphological identification using taxonomic keys [16]. All culicine larvae were discarded after counting at the sampling sites.

### Rearing and identification of anopheline mosquito’s species

All anopheline larvae samples were reared to adult mosquitoes following the methods provided by the Malaria Research and Reference Reagents Resource (MR4, BEI Resources, Virginia, USA) [17]. To maintain the same aquatic environment, the larvae were allowed to grow in the water that was collected from the field. The combination of ‘*Cerfami*’ and ‘*Bravo instant yeast’* were provided as additional food source for the larvae. Pupae were collected daily and left in paper cup until adults emerged. After emergence, male and female anophelines were sorted, counted and recorded. All adult anopheline mosquitoes were examined under dissecting microscope and morphologically identified to species complex using the identification key of Gillies and Coetzee [12].

### Habitat characterization

During the survey, environmental variables related to larval habitats were assessed. The variables recorded include habitat type, crop type, turbidity, exposure to sunlight, distance to the nearby house, vegetation, substrate types, land use and land cover. The distance to the nearest house was measured either using a tape meter when it was shorter 100 m or visually when over 100 m. Habitats exposure to sunlight was visually determined as shaded, partially shaded or sunlit. Substrate type was classified as muddy or sandy. The presence or absence of vegetation was determined visually. Vegetation type was categorized as emergent, submerse, floating, shed or mixed. Land use type was also grouped into the cultivated land/crop, grassland/pasture, wetland/swamp, road, and shrub land. Turbidity was classified as clear, turbid, and more turbid [18, 19].

Geographic coordinate readings of each surveyed aquatic habitat were recorded using Geographic Positioning System (GPS).

### Data analysis

Anopheline larvae occurrence was defined as the presence or absence of the larvae. The density of anopheline larvae was estimated as the number of larvae per dip for each habitat type. Larval abundance was calculated as a number of larvae collected in each type of habitat. Pearson chi-square analysis was applied to assess the association between anopheline mosquito larvae occurrence and environmental parameters linked to larval habitats. Multiple logistic regression analysis was used to determine primary parameters that influence the occurrence of anopheline larvae. Test of significance was done assuming *α* at 0.05 and a *P*-value less than 0.05 was considered significant. All analyses were done using Microsoft Excel (Version 2016, Microsoft Corporation, Washington, USA) and SPSS statistical software version 25 (SPSS Inc., Chicago, IL, USA).

## Results

### Mosquito larval habitat types and positivity

Overall, 319 mosquito habitats were surveyed, of which 180 (56.4%) were from irrigated area, and the remaining 139 (43.7%) were from non-irrigated area (Table 1). Habitat types included swamps/marshy (*n* = 83; 26.0%), rain pool (*n* = 75; 23.5%), stream shoreline (*n* = 31; 9.7%), spring seepage (*n* = 24; 7.5%), tire trucks/road puddle (*n* = 21; 6.6%), animal foot print (*n* = 21; 6.6%), irrigation canal 14 (4.4%), hippo trench 13 (4.1%), man-made pool 8 (2.5%), farm ditch 5 (1.6%), drainage ditch 5 (1.6%), pit 5 (1.6%), rice puddle 5 (1.6%) and other 5 (1.6%).

**Table 1:** Types of potential mosquito breeding habitats and their positivity in irrigated and non-irrigated areas, in and around Arjo-Dedessa sugar development site, southwest Ethiopia (2017–2018)

Sites	Habitat type	Number of habitat surveyed	Positive for anopheline *n* (%)	Positive for anopheline & culicine *n* (%)	Positive for anopheline alone *n* (%)	Positive for culicine alone *n* (%)
Irrigated area	Rain pool	46	26 (56.5)	21 (45.3)	5 (11.2)	15 (32.6)
	Swamp	23	13 (56.5)	10 (43.5)	3 (13.0)	6 (26.1)
	Stream shoreline	19	11 (58.0)	10 (52.6)	1 (5.3)	7 (36.8)
	Tire track/road puddle	19	14 (73.7)	10 (52.6)	4 (21.1)	3 (15.8)
	Spring seepage	16	7 (43.8)	4 (25.0)	3 (18.8)	5 (31.3)
	Hippo trench	13	4 (30.8)	3 (23.1)	1 (7.7)	8 (61.5)
	Animal foot print	10	7 (70.0)	5 (50.0)	2 (20.0)	1 (10.0)
	Earth bottom irrigation canals	14	5 (35.7)	5 (35.7)	-	8 (57.2)
	Drainage ditch	4	1 (25.0)	1 (25.0)	-	2 (50.0)
	Man-made pools	7	6 (85.7)	5 (71.4)	1 (14.3)	1 (14.3)
	Pit	3	-	-	-	3 (100.0)
	Farm ditch	2	2 (100.0)	1 (50.0)	1 (50.0)	-
	Water container	1	-	-	-	1 (100.0)
	Rice puddle	1	1 (100.0)	1 (100.0)	-	-
Non-irrigated area	Swamp	60	32 (52.5)	29 (47.5)	3 (4.9)	19 (31.1)
	Rain-pool	29	8 (27.6)	6 (20.7)	2 (6.9)	9 (31.0)
	Stream shoreline	12	7 (58.3)	6 (50.0)	1 (8.3)	2 (16.7)
	Animal foot print	11	4 (36.4)	3 (27.3)	1 (9.1)	1 (9.1)
	Spring seepage	8	1 (12.5)	-	1 (12.5)	4 (50.0)
	Man-made pools	5	3 (60.0)	2 (40.0)	1 (20.0)	2 (40.0)
	Farm ditch	3	-	-	-	2 (66.7)
	Pit	2	-	-	-	1 (50.0)
	Rock pool	1	-	-	-	1 (100.0)
	Drainage ditch	1	0	0	-	1 (100.0)
	Tire track/road puddle	2	-	-	-	-
	Rice puddle	4	-	-	-	-

-: Not applicable

Among the surveyed larval habitats, 80.6% (*n* = 257) were positive for mosquito larvae (either *Anopheles* and/or culicine) and anopheline mosquito larvae were found in 59.1% (*n* = 152) habitats (Table 1). The majority of anopheline mosquito breeding habitats were from the irrigated area (63.8%; *n* = 97) while the remaining 36.2% (*n* = 55) were from the non-irrigated area.

A total of 17 different types of mosquito breeding habitats was encountered in the irrigated area, of which 14 (83%) were positive for anopheline larvae. In the non-irrigated area, seven of the 13 (58.3%) surveyed mosquito breeding habitats were positive for anopheline larvae (Table 1). The association between the occurrence of anopheline mosquito larvae and type of agroecosystem was statistically significant (*χ*^2^ = 6.448, df = 1, *P* = 0.011).

### Anopheline larval density

Mean mosquito larval density varied significantly across different types of breeding habitats in both irrigated (ANOVA, F = 2.610, df = 13, *P* = 0.004) and non-irrigated (ANOVA, F = 2.800, df = 6, *P* = 0.02) areas during the study period. In the irrigated area, hoof prints had the highest mean larval density (3.7 larvae/dip) followed by hippo trenches (1.0 larvae/dip) and man-made pool (1.0 larvae/dip). Similarly, the highest mean larval density in the non-irrigated area was observed in hoof prints (1.7 larvae/dip) followed by rain pools (0.7 larvae/dip) and stream shoreline (0.7 larvae /dip).

There was no significant difference in mean larval density between irrigated and non-irrigated areas. Likewise, the mean larval density between dry and wet season was not significant (*P* > 0.05). However, the overall larval abundance in the irrigated area was higher by 16.6% when compared to the non-irrigated area.

### Characteristics of anopheline breeding habitats

The majority (70–71%) of anopheline breeding habitats were located within 500 m from nearby houses in the irrigated and non-irrigated areas (Table 2). About half of the mosquito breeding habitats had vegetation cover, mainly an emerging vegetation. The majority of habitats were found to be turbid in both irrigated (75.3%) and non-irrigated (61.8%) areas. Most of the anopheline mosquito breeding habitats were fully exposed to sunlight. With respect to land use types 43.6 and 40.0% of habitats were wetland/swamp and grassland/pasture, respectively (Table 2).

**Table 2:** Physical characteristics of the anopheline larvae breeding habitats from the irrigated and non-irrigated area, in and around Arjo-Dedessa sugar development site, southwest Ethiopia (2017–2018)

Physical characteristics		Sites		Total *n* (%)
		Non-irrigated area *n* (%)	Irrigated area *n* (%)	
Substrate	Muddy	54 (98.2)	97 (100.0)	151 (99.3)
	Sandy	1 (1.8)	-	1 (0.7)
Vegetation presence	No	21 (38.2)	46 (47.4)	67 (41.1)
	Yes	34 (61.8)	51 (52.6)	85 (55.9)
Vegetation type (*N* = 85)	Emergent	25 (73.5)	31 (60.7)	56 (65.8)
	Submersed	9 (26.5)	8 (15.7)	17 (20.0)
	Floating	-	2 (3.9)	2 (2.3)
	Shaded	-	5 (9.8)	5 (5.8)
	Mixed	-	5 (9.8)	5 (5.8)
Turbidity	Clear	21 (38.2)	23 (23.7)	44 (28.9)
	Turbid	19 (34.6)	47 (48.5)	66 (43.5)
	More turbid	15 (27.2)	26 (26.8)	41 (26.9
Exposure to sun	Shady	-	1 (1.0)	1 (0.7)
	Partially shady	2 (3.6)	9 (9.3)	11 (7.2)
	Sunlit	53 (96.4)	87 (89.7)	140 (92.1)
Seasonality	Permanent	5 (9.1)	24 (24.7)	29 (19.1)
	Temporal	50 (89.9)	73 (75.3)	122 (80.3)
Land use type	Shrub land	2 (3.6)	8 (8.2)	10 (6.6)
	Grassland/pasture	22 (40.0)	32 (32.9)	54 (35.5)
	Wetland/swamp	24 (43.6)	8 (8.2)	32 (21.1)
	Cultivated land/cropland	6 (10.9)	45 (46.4)	51 (33.5)
	Road	1 (1.8)	4 (4.1)	5 (3.3)
Distance from nearby house	Less than 100 m	2 (3.6)	5 (5.2)	7 (4.6)
	Between 100 m & 200 m	3 (5.5)	15 (15.5)	18 (11.8)
	Between 200 m & 500 m	33 (60.0)	47 (48.2)	80 (52.6)
	No house with in 500 m	17 (30.9)	30 (31.1)	47 (30.9)

-: Not applicable

### Seasonal anopheline larval habitat diversity

**Fig. 2:**
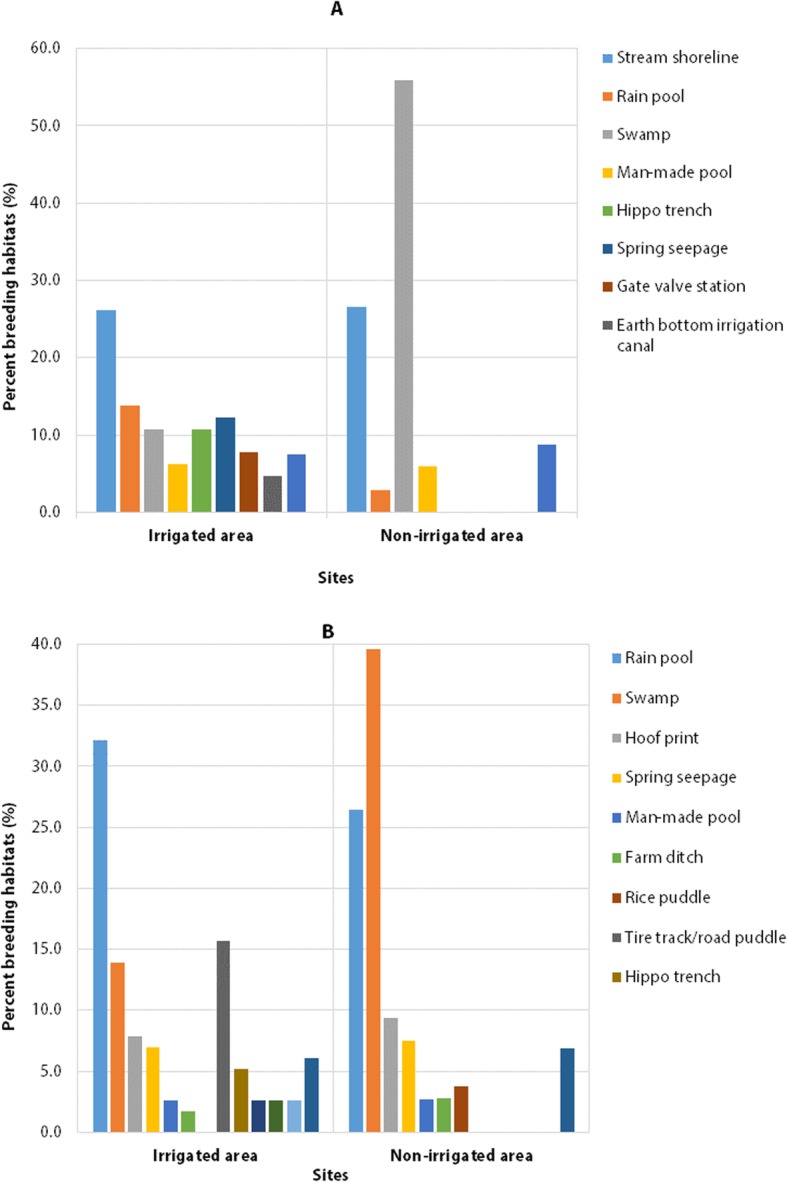
Proportion of habitat diversity in dry (A) and wet (B) seasons in the irrigated and non-irrigated areas around Arjo-Dedessa development site, Southwest Ethiopia (2017–2018)

During the dry season, stream shorelines, rain pools, swamp/marsh, spring seepages, hippo trenches and Earth bottom irrigation canals were the most frequently encountered mosquito breeding habitats in the irrigated area. In the non-irrigated area, swamps/marshes and stream shorelines were the most common larval habitats during the dry season (Fig. 2A).

During the wet season, rain pools, tire tracks/road puddles and swamps/marshes, were the predominant mosquito breeding habitats in the irrigated area; while swamps and rain pools the most commonly encountered larval habitats in the non-irrigated area (Fig. 2B).

**Fig. 3:**
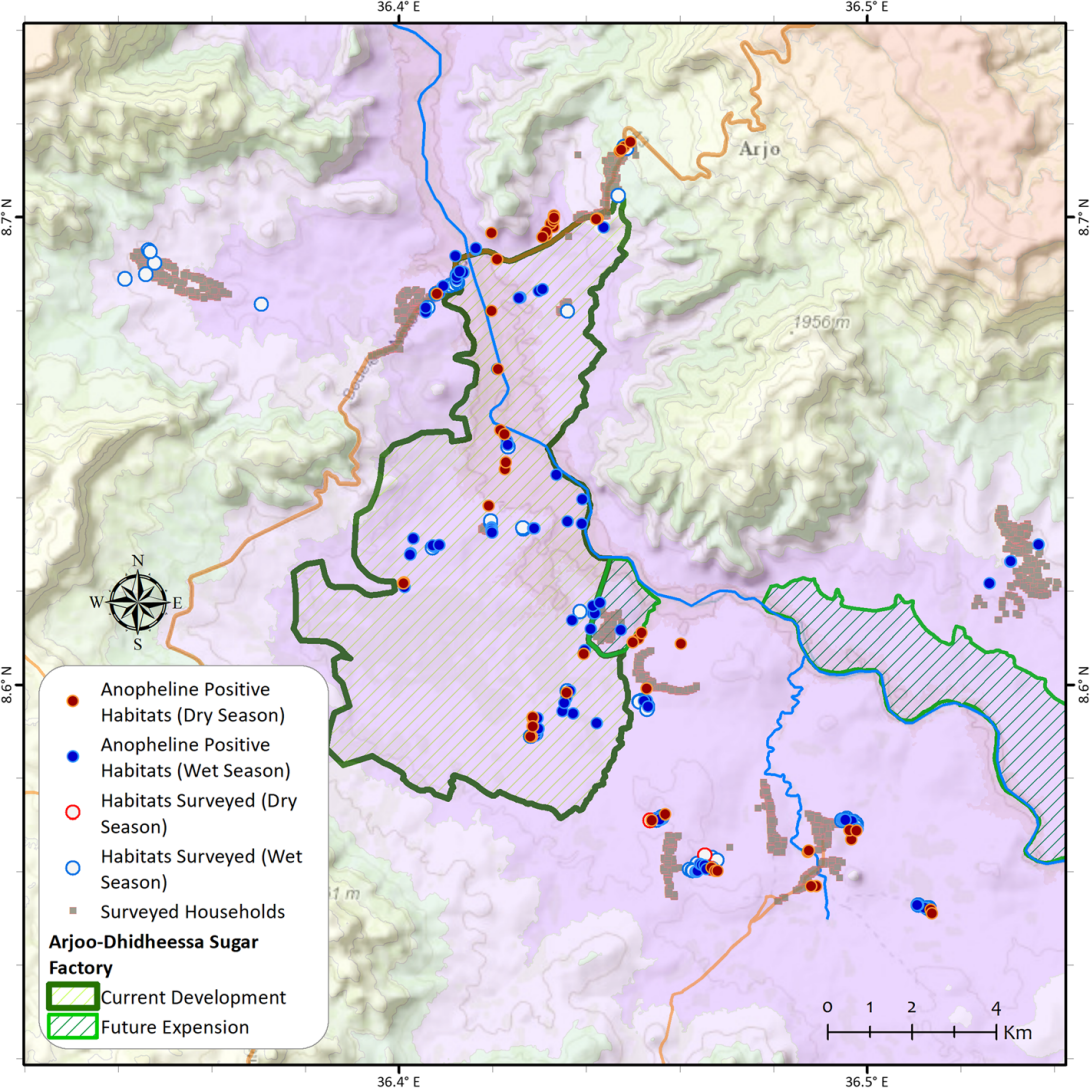
Distribution of breeding habitats positive for anopheline mosquito larvae in wet and dry seasons around Arjo-Dedessa development site, Southwest Ethiopia (2017–2018)

The association between anopheline larval occurrence and seasons was statistically significant in both irrigated (*χ*^2^ = 7.284, df = 1, *P* = 0.007) and non-irrigated area (*χ*^2^ = 11.429, df = 1, *P* = 0.001). A higher number of anopheline larval positive habitat was recorded in the wet season than dry season (Fig. 3). Generally, more diverse mosquito breeding habitats were observed in the irrigated area than the non-irrigated area during the study period.

### Anopheline larvae abundance

**Fig. 4:**
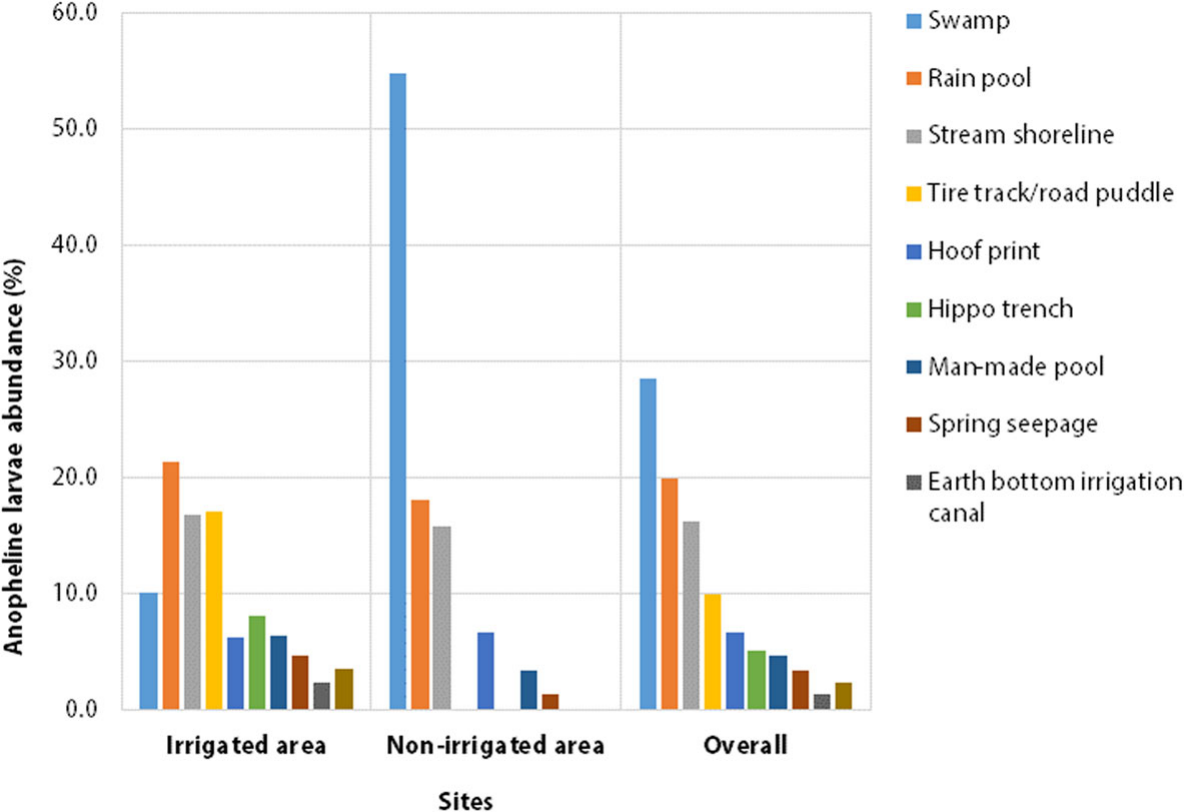
Anopheline mosquito larval abundance in irrigated and non-irrigated areas, in and around Arjo-Dedessa sugar development site, Southwest Ethiopia (2017–2018) *Others includes: used tire, rock pool, water container, natural pond and pit.

A total of 1523 anopheline larvae (1195 early, 348 late instars) and 5287 culicine were collected during the study period (Fig. 4). Out of the total anopheline larvae collected, 58.3% (*n* = 888) and 41.7% (*n* = 635) were from the irrigated and non-irrigated areas, respectively. In the irrigated area, rain pools, tire trucks/road puddles, stream shorelines and swamps were the major sources of anopheline larvae, all together accounting for 65.4% of the total larval collection. In the non-irrigated area, swamps were the most productive habitats followed by rain pool and stream shoreline, together accounting for 88.6% of the total larval samples (Fig. 4). Overall, anopheline larval abundance was generally higher in the irrigated than non-irrigated areas both during the dry and wet seasons.

**Fig. 5:**
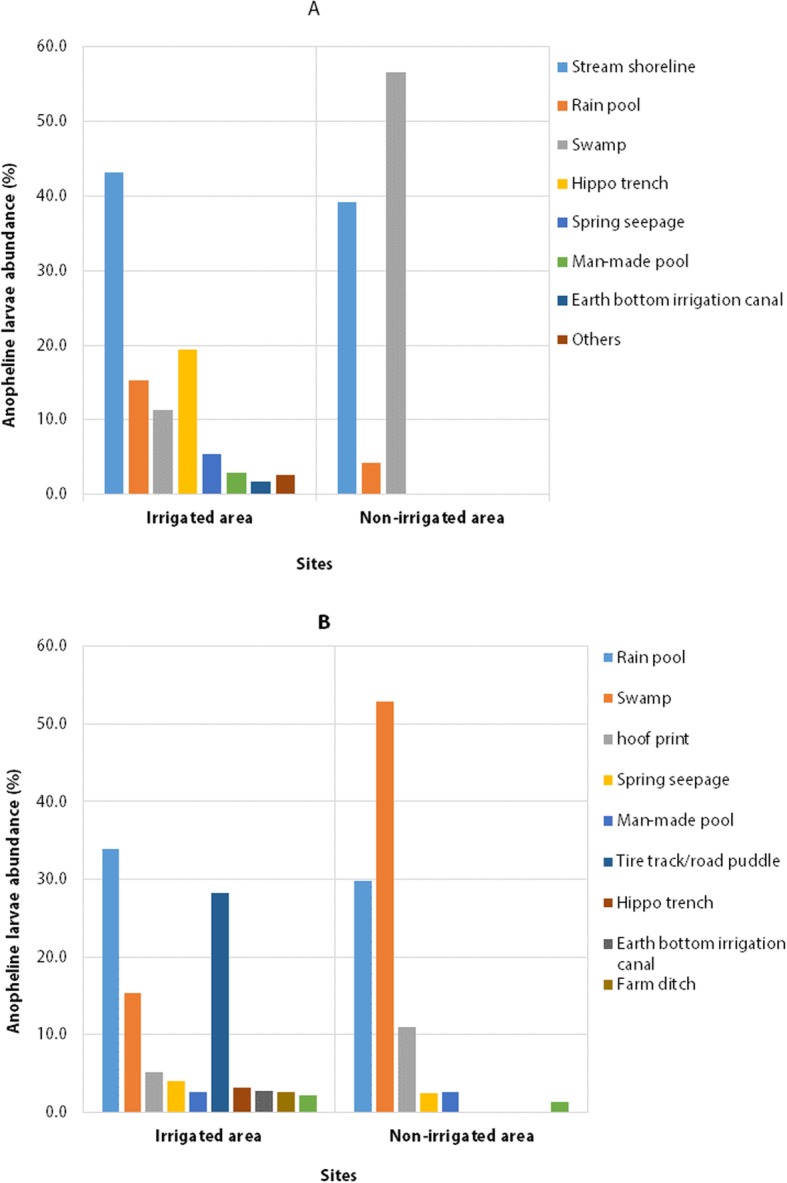
Anopheline mosquito larval abundance in irrigated and non-irrigated areas during dry (A) and wet seasons (B), in and around Arjo-Dedessa sugar development site, Southwest Ethiopia (2017–2018) *Other habitats include: gate valve station, drainage ditch and rice paddle.

In the irrigated area, anophleine larval samples were mainly collected from stream shorelines and hippo trenches during the dry season and from rain pools and tire tracks/road puddles during the wet season (Fig. 5). In the non-irrigated area, swamps were major sources of anopheline larvae both during the wet and dry seasons. Overall, a higher abundance of anopheline larvae was noted in the irrigated than non-irrigated areas during the study period.

### Association between environmental parameters and anopheline mosquito’s larvae occurrence

Results of Pearson’s chi-square analysis showed a significant association between anopheline larvae occurrence and environmental parameters, season (*χ*^2^ = 63.122, df = 1, *P* < 0.001), agroecosystem (being irrigated or non-irrigated) (*χ*^2^ = 6.448, df = 1, *P* = 0.011), and turbidity (*χ*^2^ = 7.296, df = 2, *P* = 0.025). Multiple logistic regressions indicated that agroecosystem type was the primary predictor for anopheline mosquitoes larval occurrence (*OR* = 1.844, 95% *CI*: 1.153–2.949, *P* = 0.011) (Additional file 1: Table S1).

#### *Anopheles* mosquito species composition

**Fig. 6:**
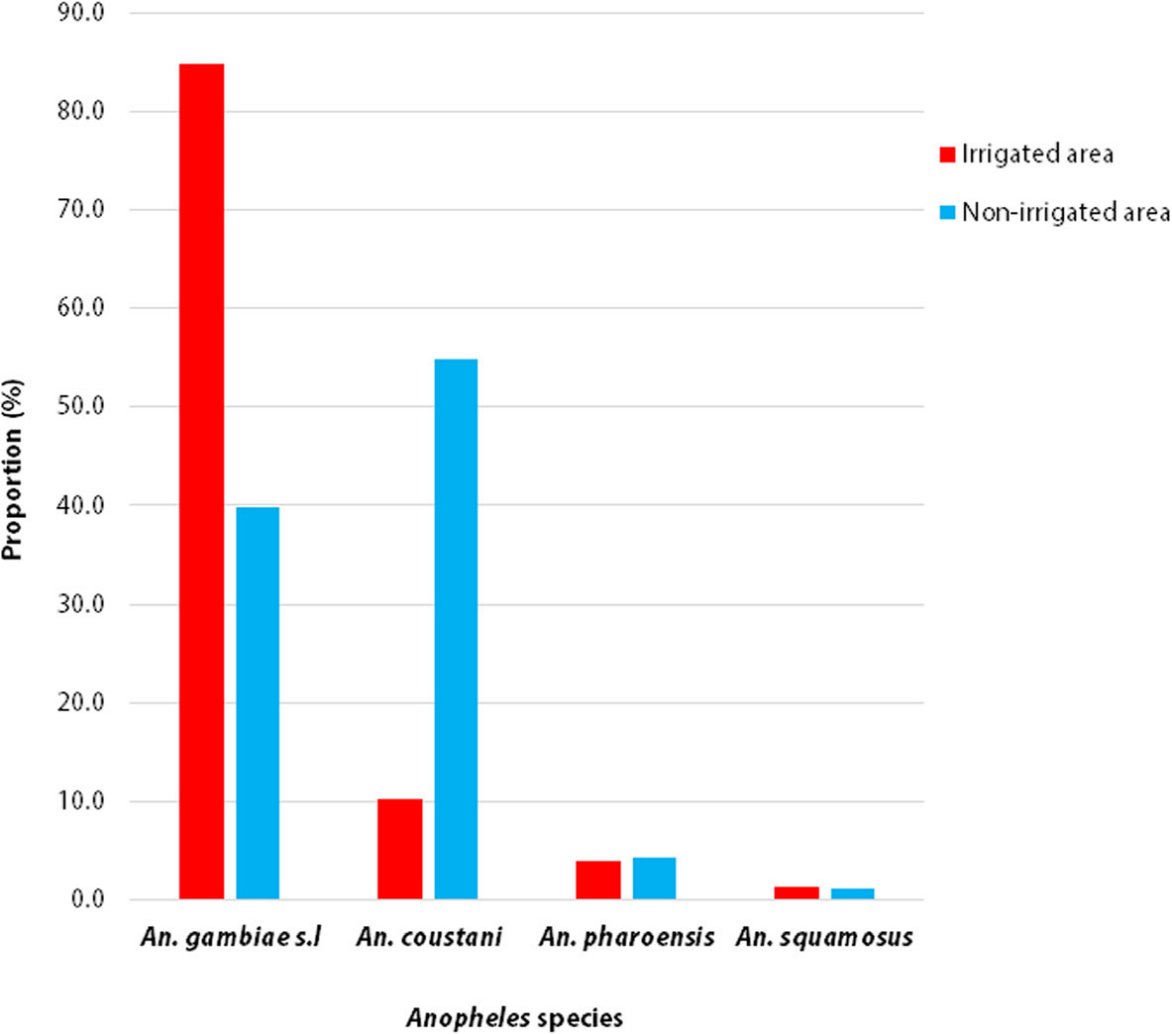
Distribution of adult female anopheline mosquito species in irrigated and non-irrigated areas, in and around Arjo-Dedessa sugar developmental site, Southwestern Ethiopia (2017–2018)

About half (*n* = 755; 49.6%) of the anoheline larval collections reared were emerged to adults, of which 349 were females and 406 were males (Fig. 6). The majority (73%) of them were from the irrigated area. Overall, four *Anopheles* species (*Anopheles gambiae* s.l.*, An. coustani, An. pharoensis,* and *An. squamosus*) were recorded. In the irrigated area, *An. gambiae* s.l. was the predominant species (84.8%) followed by *An. coustani* (10.0%), whereas in the non-irrigated setting, *An. coustani* (54.8%) was the most common species followed by *An. gambiae* s.l. (39.8%) (Fig. 6).

## Discussion

The study revealed that the anopheline mosquito breeding habitats were diverse in the irrigated areas. The diversity of mosquito breeding habitats in the irrigated area was two-fold higher than the non-irrigated area, indicating that the irrigation development contributed to the proliferation of malaria mosquito breeding habitats. Improper ground excavation, frequent vehicles and machineries movements during planting and harvesting, lack of maintenance and poor environmental management contributed to the formation of numerous mosquito breeding habitats in the irrigation project area as noted elsewhere in Africa [5, 20, 21]. Similarly, several studies elsewhere in Africa have suggested that changes in land use have influenced malaria vector larval habitat availability and distribution [22, 23]. The findings from the present study are also in agreement with previous studies in central Ethiopia where a higher larval and adult abundance of the malaria vectors was recorded in the irrigated than non-irrigated villages [24]. A study conducted in western Ethiopia reported that higher malaria prevalence and transmission risk increased due to high vector abundance in the irrigated sugarcane agroecosystem than non-irrigated agroecosystem [21]. Generally, an increase in mosquito breeding habitats results an increase in vector density and eventually leading to increased malaria transmission [25, 26].

Most of the mosquito breeding habitats identified in this study were previously reported elsewhere [13, 27, 28]. However, the nature and formation of some of the habitats made them specific and unique to the study area and thus can be target for intervention. For instance, the mosquito habitat like hippo-trench was specific to the irrigated area. Hippo-trenches were deep excavation, around 2 m, canal structures designed to prevent the hippos from entering into the sugarcane farm. The trenches were situated at the periphery of the farm and designed to collect water from surrounding streams or springs (Additional file 2: Figure S1). During the rainy season, the trenches remained filled with water but became shallow and conducive for mosquito breeding during the dry season. A study conducted in Kenya suggested that habitat size is an important determinant of habitat stability and mosquito occurrence [29]. Identifying vector breeding habitats is important to target them for larval management.

In the irrigated area, rain pools, tire tracks/road puddles and swamps were found to be the major breeding habitats for *Anopheles* mosquitoes during the wet season, while stream shoreline and hippo-trenches provided larval breeding grounds during the dry season. On the other hand, in the non-irrigated area, swamps and rain pools were the major larval breeding habitats during the wet season, while swamps and stream shorelines were common breeding grounds during the dry season. This showed that targeting these habitats through larval management could help significantly reduce the vector mosquito population abundance and eventually reduces malaria transmission intensity in the area. In Africa, larval source management have been shown to be very effective in areas where mosquito breeding habitats are distinct and accessible [30]. Studies showed that when larval management is integrated with LLINs and IRS, a great improvement would be seen in malaria control efforts than IRS and LLINs alone [31, 32]. The present study indicated that availability of distinct mosquito breeding habitats during the dry and wet seasons, indicating the potential use of larval source management to reduce the mosquito population.

The difference in *Anopheles* larval occurrence between the irrigated and non-irrigated areas could partly be due to the differences in the microclimate in two agroecosystems. About two-third of *Anopheles* positive breeding habitats were found to be turbid. A study conducted in Ethiopia reported that *An. arabiensis*, the major malaria vector in the country, lays more eggs in the turbid water proximity to pollen-shedding maize farms than clear water [33]. The possible explanation for preference of turbid water over clear might be due to difference in soil nutrients that influence the enrichment of bacteria that serve as a food source of larvae, and possibly as oviposition attractants [34].

This study had several limitations. The study did not include data of microclimate variation between the two agroecosystems. The variation in microclimate may have an influence on mosquito larval habitat productivity. Furthermore, use of pesticides might contribute to insecticide resistant mosquitos’ abundance and hence affecting the ongoing malaria control using IRS and LLINs. Future research is therefore needed to better understand of the effect of environmental modification on the insecticide resistance status of vector mosquitoes and their survivorship.

## Conclusions

*Anopheles* mosquito breeding habitat diversity, positivity and abundance were found to be higher in the irrigated than non-irrigated areas during the dry and wet seasons. The findings of this study suggest that irrigation development activities amplify the proliferation of aquatic breeding habitats for malaria vector mosquitoes that may lead to higher risk of malaria transmission. Identifying major malaria vector breeding habitats helps devise tailor-made interventions such as larval source management to reduce the risk of malaria around irrigation schemes.

## Supplementary information

**Additional file 1 **: **Table S1** Logistic regression analysis for anopheline larvae occurrence, around Arjo-Dedessa sugar development site, southwest Ethiopia (2017–2018)

**Additional file 2 **: **Figure S1** Hippo-trenches at the edge of the sugarcane farm to prevent the Hippos from entering into sugarcane farm, Arjo-Dedessa sugar developmental site, Southwestern Ethiopia (2017–2018)

## Data Availability

The datasets used and/or analysed during the current study are available from the corresponding author on reasonable request.

## Abbreviations

*CI*: Confidence interval
IRS: Indoor residual spray
ITNs: Insecticide-treated nets
LLINs: Long-lasting insecticidal nets
LSM: Larval source management
*OR*: Odds ratio
