# Supplementary material for: Effects of environmental modification on the diversity and positivity of anopheline mosquito aquatic habitats at Arjo-Dedessa irrigation development site, Southwest Ethiopia
Source: Infect Dis Poverty. 2020 Jan 27;9:9. doi: 10.1186/s40249-019-0620-y (PMC6986026; doi:10.1186/s40249-019-0620-y)
Supplement: Supplementary file 1 — Additional file 1 : Table S1 Logistic regression analysis for anopheline larvae occurrence, around Arjo-Dedessa sugar development site, southwest Ethiopia (2017–2018) [file 40249_2019_620_MOESM1_ESM.docx]

| Variable | B | S.E. | Wald | df | Sig. | OR (95%CI) |
| --- | --- | --- | --- | --- | --- | --- |
| Site | 0.612 | 0.240 | 6.528 | 1 | 0.011 | 1.844 (1.153 - 2.949) |
| Habitat turbidity | -0.165 | 0.106 | 2.438 | 1 | 0.118 | 0.848 (0.689 - 1.043) |
| Season | -0.322 | 0.257 | 1.571 | 1 | 0.210 | 0.725 (0.438 - 1.199) |
| Constant | 0.748 | 0.516 | 2.102 | 1 | 0.147 |  |
