# Supplementary figures and images for: Effects of environmental modification on the diversity and positivity of anopheline mosquito aquatic habitats at Arjo-Dedessa irrigation development site, Southwest Ethiopia
Source: Infect Dis Poverty. 2020 Jan 27;9:9. doi: 10.1186/s40249-019-0620-y (PMC6986026; doi:10.1186/s40249-019-0620-y)

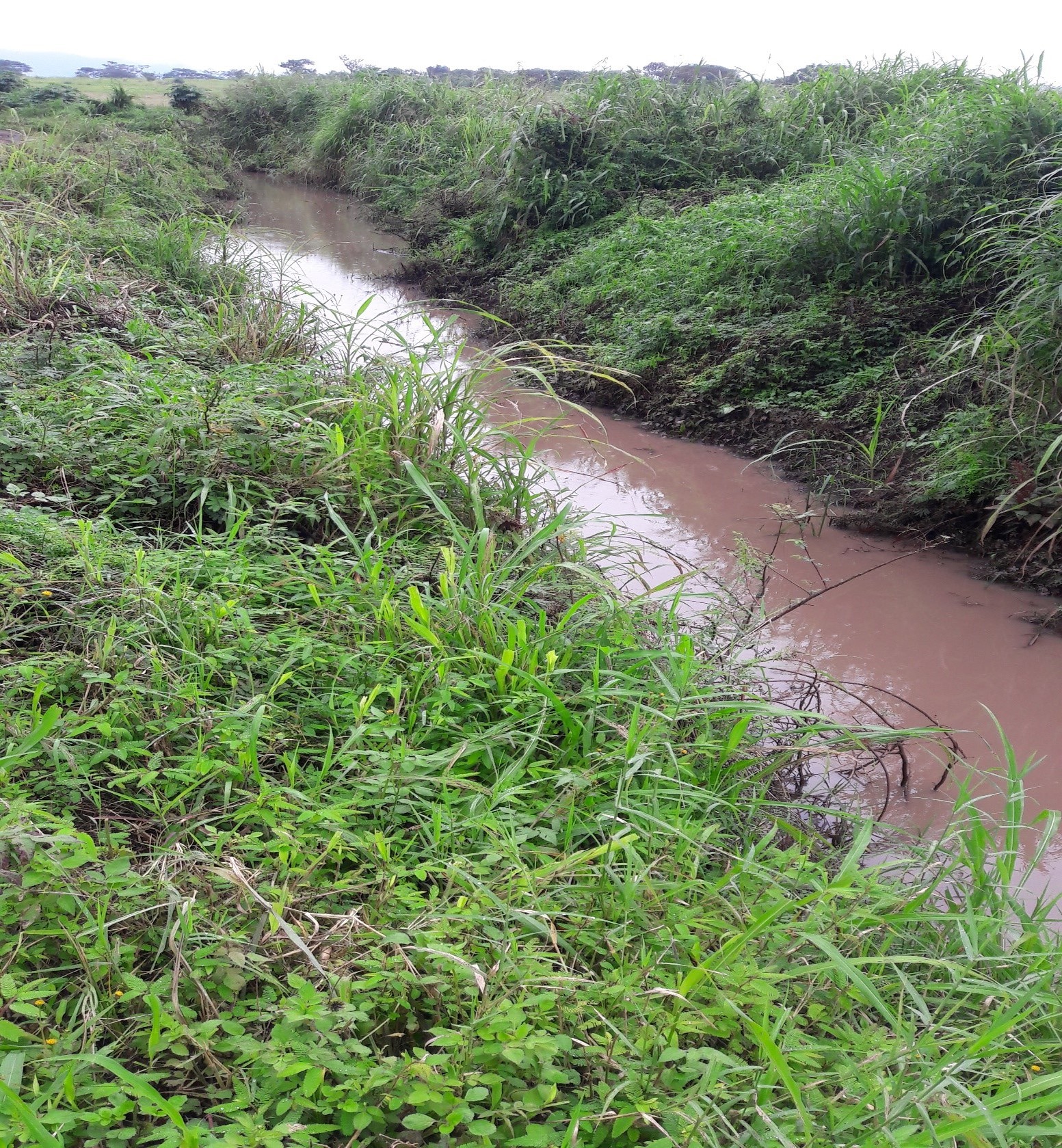

Supplement: Supplementary file 2 — Additional file 2 : Figure S1 Hippo-trenches at the edge of the sugarcane farm to prevent the Hippos from entering into sugarcane farm, Arjo-Dedessa sugar developmental site, Southwestern Ethiopia (2017–2018) [file 40249_2019_620_MOESM2_ESM.jpg]
